# A Systematic Review Evaluating the Surgical Management of Cervical Spine Fractures in Patients With Ankylosing Spondylitis

**DOI:** 10.7759/cureus.99991

**Published:** 2025-12-24

**Authors:** Shahswar Arif, Zarina Brady, Reagan O'Kane, Ciaran Doherty, Christopher McKee, Jagdeesh Nijjher, Rakesh Dhokia, Nikolay Peev

**Affiliations:** 1 Trauma and Orthopaedics, Altnagelvin Hospital, Londonderry, GBR; 2 Surgery, Western Health and Social Care Trust, Londonderry, GBR; 3 Medicine, Queen's University, Belfast, GBR; 4 Orthopaedics, Musgrave Park Hospital, Belfast, GBR; 5 Trauma and Orthopaedics, Royal Victoria Hospital, Belfast, GBR; 6 Neurosurgery, Belfast Health and Social Care Trust, Belfast, GBR

**Keywords:** ankylosing spondylitis, cervical spinal fractures, intraoperative complications, mortality rate, postoperative complications

## Abstract

Patients with ankylosing spondylitis (AS) are vulnerable to cervical spinal fractures despite minute trauma. As there is a lack of consensus on which surgical approach is the most efficacious in treating cervical spinal fractures in AS patients, we sought to compare the posterior-only and combined (anterior-posterior) approach regarding intraoperative and postoperative complications. This systematic review was registered with the International Prospective Register of Systematic Reviews (PROSPERO) database (ID: CRD42024284877). Studies published up to May 20, 2025, were searched in PubMed, MEDLINE, and CENTRAL (Cochrane Central Register of Controlled Trials) with predetermined terms. Studies reporting outcomes, including intraoperative and postoperative complications and mortality rate, were evaluated. Included studies underwent a risk of bias assessment. Seventeen studies (one prospective, 16 retrospective) involving 858 patients (with AS and cervical spine fractures) with an average age of 59.9 years were included. The most common mechanism of injury was minor trauma (173, 66%), and C6-7 was the most common level injured (185, 50.6%). Although the posterior-only approach yielded fewer intraoperative complications (4 (4.8%) vs. 2 (15%); p>0.05), postoperative complications (17 (7.5%) vs. 16 (22.6%); p>0.05), and instrument-related complications (1 (2.8%) vs. 3 (12.5%); p>0.05) compared to the combined approach, the difference was not statistically significant. However, the combined approach recorded a significantly lower mortality rate (3 (9.2%) vs. 13 (24.5%); p>0.05) when compared with the posterior-only approach. The results of this systematic review suggest that both the combined and posterior-only approaches have advantages and disadvantages and approach selection should be done on a case-by-case basis. Future randomized controlled trials directly evaluating the efficacy of surgical approaches in such patients are needed to form a consensus.

## Introduction and background

Ankylosing spondylitis (AS) is an autoimmune condition involving the spine, sacroiliac joints, and entheses [1]. The cervical spine, followed by the thoracolumbar region, is the most frequent spinal fracture site in AS patients [1,2]. There is often a delay in diagnosis, as the symptoms are not very overt [1-3]. Standard imaging is not optimal for noticing the osteoporosis-linked shearing fractures, particularly in the spine. Even with the slightest fracture suspicion, computed tomography (CT) and magnetic resonance imaging (MRI) should be recommended [4-6]. Neurological deficit can occur at the time of the fracture or upon its displacement, specifically in hyperextension injuries [7]. Surgery is indicated in cases with unstable fractures and severe neurological deficit [5,6,8]. The goal is to prevent complications linked with nonoperative measures [9]. There is still no consensus on the best approach to treat AS-linked cervical spinal fractures. Thus, we performed a systematic review of randomized controlled trials and prospective and retrospective studies to evaluate the postoperative neurological function and perioperative complications in surgically treated AS patients with cervical spinal fractures.

## Review

**Figure 1:**
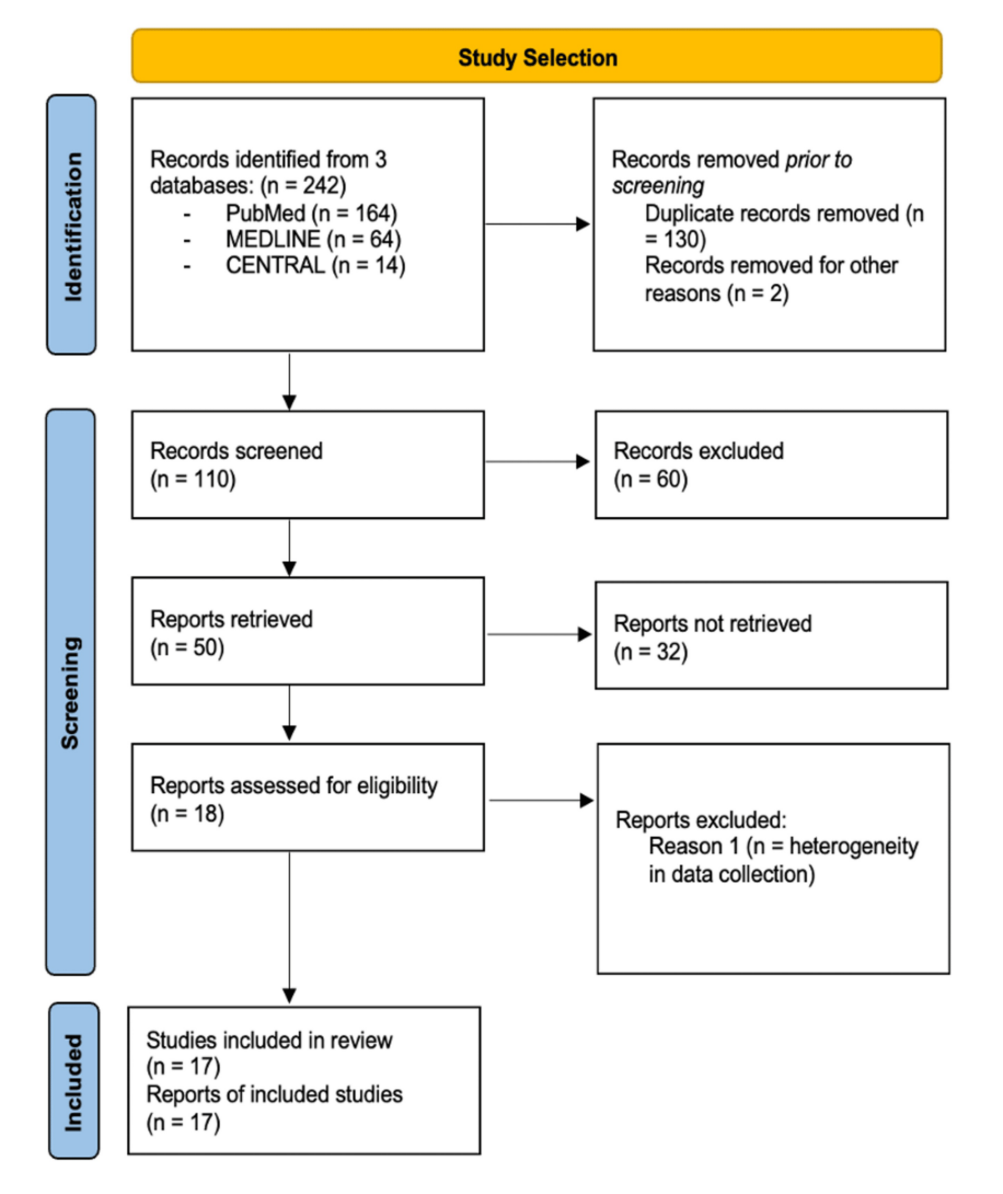
PRISMA flowchart for study selection PRISMA: Preferred Reporting Items for Systematic Reviews and Meta-Analyses; CENTRAL: Cochrane Central Register of Controlled Trials

This systematic review was registered with the International Prospective Register of Systematic Reviews (PROSPERO) database (ID: CRD42024284877). The search strategy in the published protocol included studies reporting neurological evaluation and perioperative complication rate comparing surgical approaches (posterior-only or combined approach) in AS patients with cervical spinal fractures. Inclusion criteria regarding the study design included randomized controlled trials (RCTs), retrospective studies, and prospective studies. In addition, only studies that evaluated AS patients with cervical spine fractures were considered for inclusion. For quantitative analysis, intraoperative complications such as epidural haematoma and incidental durotomy were evaluated, instrument-related complications such as screw loosening/loss of fixation were evaluated, and postoperative complications such as dysphagia, pneumonia, urinary tract infection, and deep vein thrombosis were included. A detailed search of studies published on PubMed, MEDLINE, and CENTRAL (Cochrane Central Register of Controlled Trials) up to May 20, 2025, was carried out. The search strategy included keywords ("Ankylosing Spondylitis", "Cervical Spine Fractures", "Peri-operative Complications", "Spinal Fractures") to identify potential articles. Figure 1 illustrates the study selection process using the Preferred Reporting Items for Systematic Reviews and Meta-Analyses (PRISMA) flowchart [10].

The search strategy was compiled in consultation with neurosurgical and orthopaedic surgeons, and no language or publication date restrictions were used. Titles as well as summaries of all studies underwent evaluation by authors independently, followed by the full-text screening of any studies that warranted inclusion. Conflicts were resolved by two senior co-authors (RD, NP). Authors used a standardized data extraction form to collate the study design, publication year, gender and age of patients, body mass index, mechanism of injury (major trauma/falls), fracture level (cervical), surgical approach (posterior-only, combined), intraoperative complications (epidural haematoma, iatrogenic durotomy), instrument-related complications (screw loosening/loss of fixation), postoperative complications (dysphagia, pneumonia, urinary tract infection, deep vein thrombosis), length of hospital stay, and mortality rate. Studies published in duplicate were only included once. All included studies underwent a risk of bias assessment, shown in Table 1 [9,11-26]. Studies that did not include the anterior or posterior or combined approaches were not included; studies that were not published in English or case reports were not included either.

**Table 1:** Assessment of bias +: low risk; ?: moderate risk; x: serious risk; !: critical risk

Study	Year	Bias due to confounding variable	Bias-participant selection	Bias in the classification of interventions	Bias due to deviation from the intended intervention	Bias due to missing data	Bias in measurement outcomes	Bias in the selection of reported unit	Overall bias
Alhashash et al. [11]	2023	+	?	+	+	+	+	+	+
Shetty et al. [12]	2023	+	?	+	+	?	+	+	?
Sharma et al. [13]	2022	+	?	+	+	?	+	?	?
Tang et al. [14]	2022	?	?	+	+	+	+	+	?
Liu et al. [15]	2021	+	?	+	+	+	?	+	+
Ren et al. [16]	2021	+	?	+	+	+	?	?	?
Altun and Yuksel [17]	2016	+	?	+	+	+	+	?	+
Lukasiewicz et al. [18]	2016	+	?	+	+	+	+	?	+
Robinson et al. [19]	2015	?	?	+	+	+	?	?	?
Schiefer et al. [20]	2015	?	?	+	+	x	+	?	x
Robinson et al. [21]	2015	?	?	+	+	+	+	+	?
Mathews and Bolesta [22]	2013	?	?	+	+	+	?	+	?
Kouyoumdjian et al. [23]	2012	?	?	+	+	+	?	+	?
Lv et al. [24]	2009	x	!	+	+	+	?	?	x
Sapkas et al. [25]	2009	+	?	+	+	+	+	+	+
Taggard and Traynelis [9]	2000	?	?	+	+	x	+	!	x
Olerud et al. [26]	1996	?	?	+	+	+	?	+	?

Results

The preliminary search resulted in 242 original articles after the removal of duplicates, of which 110 articles were deemed potentially eligible. A total of 17 studies were included in the final quantitative analyses. Characteristics of the included studies are summarized in Table 2. The relevant 17 studies published between 1983 and 2023 were included.

**Table 2:** Patient characteristics AS: ankylosing spondylitis AO:

Characteristics	AS patients with spinal fracture cohort
Study design (n)	
Prospective	1
Retrospective	16
N	858
Average age (years)	59.9
Mechanism of injury, n (%)	
Major trauma (motor vehicle accidents, assaults/crush)	43 (25%)
Falls	173 (66%)
Fracture level, n (%)	
C1	2 (2.9%)
C2	5 (5.2%)
C4	3 (3.2%)
C5	4 (3.4%)
C6/C7	185 (50.6%)
C7	10 (8%)
C7/T1	18 (9%)
Fracture type, n (%)	
A	-
B1	-
B2	19 (13%)
B3	60 (54%)
C	35 (31.1%)
Preoperative neurological deficit, n (%)	168 (77.7%)

Primary Outcomes

For the posterior-only cohort, the overall intraoperative complication rate was four (4.8%), with incidental durotomy and epidural haematoma occurring in two (3.7%) and two (5.8%) patients, respectively. In the combined (anterior-posterior) approach group, the overall intraoperative complication rate was two (15%), with incidental durotomy and epidural haematoma occurring in one (10%) and one (20%) patient, respectively (Table 3). The instrument-related complication rate was lower in the posterior-only cohort (1, 2.8%) than in the combined approach group (3, 12.5%) (Table 4). Postoperative complication rate was higher in the combined approach cohort (16, 22.6%) in comparison to the posterior-only approach group (17, 7.5%). Frequently reported postoperative complications for the combined and posterior-only approaches were pneumonia (3 (30%) vs. 6 (8.8%)), wound infection (2 (8.3%) vs. 5 (12%)), and pulmonary complications (3 (30%) vs. 4 (7.7%)) (Table 3). As regards the mortality rate, the posterior-only cohort had a higher mortality rate (13, 24.5%) in comparison to the combined approach cohort (3, 9.2%) (p<0.05) (Table 4).

**Table 3:** Comparative evaluation of intraoperative and postoperative complications between posterior-only and combined approaches EBL: estimated blood loss; DVT: deep vein thrombosis

Characteristics	Posterior-only approach	Combined (anterior-posterior) approach
Surgical details		
EBL (ml)	306.38	458.33
Operation time (mins)	161.11	213.67
Intraoperative complications, n (%)		
Epidural haematoma	2 (5.8%)	1 (20%)
Durotomy	2 (3.7%)	1 (10%)
Screw loosening/loss of fixation	1 (2.8%)	3 (12.5%)
Postoperative complications, n (%)		
Wound infection	5 (12%)	2 (8.3%)
Pulmonary complications	4 (7.7%)	3 (30%)
Dysphagia	0 (0%)	4 (27.5%)
Pneumonia	6 (8.8%)	3 (30%)
Urinary tract infection	0 (0%)	2 (20%)
DVT	2 (16.7%)	2 (20%)
Mortality rate, n (%)	13 (24.5%)	3 (9.2%)

**Table 4:** Summative evaluation between the combined and posterior-only approaches

Characteristics	Posterior-only approach	Combined approach	P-value
Intraoperative complications, n (%)	4 (4.8%)	2 (15%)	>0.05
Postoperative complications, n (%)	17 (7.5%)	16 (22.6%)	>0.05
Instrumented-related complications, n (%)	1 (2.8%)	3 (12.5%)	>0.05
Mortality rate, n (%)	13 (24.5%)	3 (9.2%)	>0.05

Discussion

This systematic review evaluated the posterior-only and combined (anterior-posterior) approaches to determine a conclusion on which approach is efficacious in treating spinal fractures in AS patients. With the increasing elderly population, the number of geriatric patients is also increasing. Patients aged above 80 years were reported to be an independent risk factor for postoperative complications [27,28].

In our systematic review, low-energy trauma was the main mechanism of spinal fractures in AS patients. This matches the trend observed in literature, in which falls were noted to be the most frequent injury mechanism [29]. Research has shown that AS is a contributing factor in low-energy traumatic spinal fractures [20]. The positive sagittal alignment itself is an independent risk factor for spinal fractures [30]. The majority of acute fractures of the spine in AS cohorts occur in the cervical region, especially at C6-C7 [31]. 

Posterior-only and combined approaches are the most frequently used techniques for subaxial spinal fractures [31-35]. Potential limitations regarding the posterior-only approach relate to posterior screw placement owing to the absence of osteological landmarks in AS [36]. Another drawback of the posterior-only approach in AS cases relates to unstable cervical spine fractures and deformities linked to the positioning of the patient. The majority of patients have a degree of kyphotic alignment with an extension-type injury. Surgeons could take into account placing the patient on a frame that allows controlled kyphosis, such as through a Wilson-type frame [7]. The combined approach gives the most construct stability [37,38].

AS patients are more susceptible to developing a neurological function deficit after spinal trauma. The literature reported neurological deficit in half of the patients [25]. Such results showed that cervical fractures in AS patients can result in severe neurological function deficit. Moreover, AS patients are susceptible to secondary neurological function deficit owing to unstable fracture configuration between fused segments [39]. Luksanapruksa et al. reported improvement in neurological function (American Spinal Injury Association (ASIA) scale) in the majority of patients in posterior-only and combined (anterior-posterior) approaches; however, two patients from the posterior-only cohort had worsening of neurological function, while no patients from the combined group worsened neurologically [40]. Literature evaluating direct comparative analyses between posterior-only and combined approaches (in such cases) is scarce [41]. The most common intraoperative complication recorded in our review was epidural haematoma. The most common instrument-related complications included were screw loosening and loss of fixation. Olerud et al. reported that fixation loss was significantly more common in one-side-only surgical approaches [26]. Due to the weakened spinal anatomy and the fact that the spine only moves at the site of the fracture, a single-side-only fixation will not withstand the stress forces focused on the internal fixation. In terms of postoperative complication rate, the posterior-only approach yielded a lower complication rate. In our review, pneumonia was one of the most commonly reported postoperative complications in both approaches [42]. An increased incidence of pulmonary diseases is observed in AS cases owing to the restrictive ankylosis of the thoracic cage [43]. Such results can help surgeons in choosing surgical approaches in AS patients with spinal fractures [42]. Momeni et al. reported that upper lobe fibrosis, interstitial pulmonary disease, and sleep apnoea are linked to AS. As the respiratory system is at a higher risk in severe AS cases, cardiopulmonary function should be taken into account when managing spinal fractures in AS patients [44].

In our systematic review, the combined approach yielded a lower mortality rate in comparison to the posterior-only approach. The risk of mortality is higher in AS patients with cervical fractures in comparison to thoracolumbar fractures. Death frequently occurred in patients with postoperative complications, especially when the pulmonary function was compromised, such as pneumonia, pulmonary fibrosis, pulmonary embolism, and acute respiratory distress syndrome [41,45].

The strengths of this systematic review are as follows: no systematic review directly evaluates surgical approaches regarding perioperative neurological improvement and perioperative complications, and the systematic review is timely and will be valuable to surgeons choosing approaches when treating cervical spinal fractures in AS patients. The limitations of this review are the following: the number of studies included is limited. There is an absence of standardized surgical procedures for the treatment of cervical fractures in AS patients. Another drawback of this relates to the wide range of scores relating to the neurological function evaluation, so several studies had to be excluded from quantitative analysis. No randomized controlled trials have been conducted on this study, resulting in a low quality of the included studies.

## Conclusions

The results of this systematic review suggest that both combined and posterior-only approaches have advantages and disadvantages and approach selection should be done on a case-by-case basis. Surgical intervention involving open reduction and internal fixation prevents worsening and improves neurological function in AS patients with cervical fractures. The combined approach yielded superior postoperative neurological outcomes as well as mortality rates in comparison to the posterior-only approach. However, the posterior-only approach yields superior outcomes with regard to intraoperative, instrument-related, and postoperative complications. Future randomized controlled trials evaluating different surgical approaches reporting objective measures are needed to form a consensus.
